# DNA-Nanostructure-Guided Assembly of Proteins into Programmable Shapes

**DOI:** 10.1021/acs.nanolett.3c04497

**Published:** 2024-01-26

**Authors:** Qinyi Lu, Yang Xu, Erik Poppleton, Kun Zhou, Petr Sulc, Nicholas Stephanopoulos, Yonggang Ke

**Affiliations:** † Department of Chemistry, Emory University, Atlanta, Georgia 30322, United States; ‡ Biodesign Center for Molecular Design and Biomimetics, Arizona State University, Tempe, Arizona 85287, United States; § School of Molecular Sciences, Arizona State University, Tempe, Arizona 85287, United States; ∥ Department of Biomedical Engineering, Georgia Institute of Technology and Emory University, Atlanta, Georgia 30322, United States

**Keywords:** DNA origami, oligomeric protein complex, DNA−protein conjugation, programmable assembly, strand displacement

## Abstract

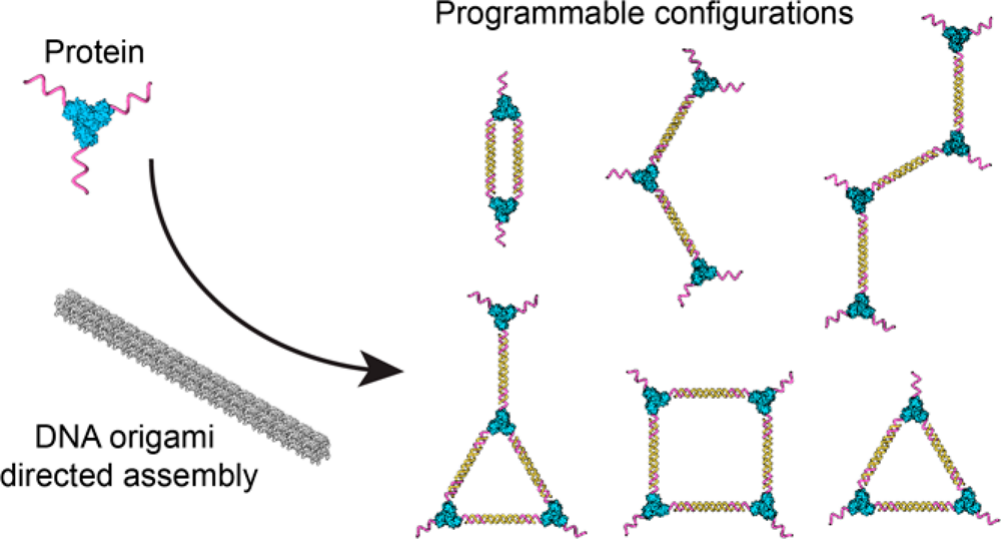

The development of methods to synthesize artificial protein complexes with precisely controlled configurations will enable diverse biological and medical applications. Using DNA to link proteins provides programmability that can be difficult to achieve with other methods. Here, we use DNA origami as an “assembler” to guide the linking of protein–DNA conjugates using a series of oligonucleotide hybridization and displacement operations. We constructed several isomeric protein nanostructures, including a dimer, two types of trimer structures, and three types of tetramer assemblies, on a DNA origami platform by using a C3-symmetric building block composed of a protein trimer modified with DNA handles. Our approach expands the scope for the precise assembly of protein-based nanostructures and will enable the formulation of functional protein complexes with stoichiometric and geometric control.

Cells comprise many modular supramolecular complexes, often made up of proteins, that perform diverse biological roles. The assembly of proteins into defined oligomeric structures allows for functions such as structural actuation in muscles,^1^ transport along the membrane via ion channels,^2^ and multienzyme catalysts.^3^ Synthetic protein assemblies are therefore a promising class of biomaterials that can mimic, or potentially surpass, the uses of naturally occurring protein complexes.^4,5^ Proteins in such complexes are normally cohered by noncovalent protein–protein interactions,^6,7^ including hydrogen bonding, electrostatic interactions, van der Waals forces, or the hydrophobic effect.^8,9^ However, these protein–protein interactions require physical contacts between two or more molecules with high specificity and a high degree of orthogonality. Although protein design approaches have shown great promise in engineering specific protein–protein interfaces (e.g., by using covalent “tags” to form oligomeric complexes)^10−14^ or *de novo* computational engineering of specific interfaces,^15−21^ it is still difficult to design highly complex and anisotropic protein assemblies.

One way to circumvent this limitation is to use protein–DNA conjugates to assemble higher-order structures.^22^ The specificity is mediated by the DNA strands covalently linked to proteins (which affords hundreds or even thousands of sequence-defined orthogonal interactions^23,24^) rather than the protein surface.^25^ Strategies for DNA-directed protein assembly can be generally divided into two categories. The first strategy places single-stranded (ss) DNA-modified proteins at designated positions on DNA nanostructures (using complementary DNA “docking” handles) to achieve controlled assembly.^26−29^ In recent years, DNA origami^30−33^ has been widely used for organization of proteins with this approach, in which the origami nanostructure is an integral component of the final product. However, the massive size of DNA origami can limit the design and function of the assembled protein complexes. The second strategy relies on connecting the oligonucleotide handles on proteins, either through attaching directly complementary strands to the proteins or introducing additional DNA connectors. Although this approach can produce more compact protein assemblies with a limited amount of DNA, it requires the synthesis of site-specific protein–DNA conjugates. For relatively simple oligomers (e.g., dimers, linear trimers) or periodic 1D^34,35^ or 3D structures,^36−38^ such synthesis is generally feasible. Nevertheless, the scalability of this strategy is questionable for more complex oligomers since increasing the number of proteins and DNA strands will inevitably lead to incorrect assemblies. To achieve more complex structures, proteins must be modified by multiple, orthogonal DNA strands (Figure 1A), which in turn requires multiple site-specific reactions and purification of the desired conjugates. Such a task not only is synthetically challenging but also decreases the yield of the final building blocks due to incomplete reaction and losses during purification.

**Figure 1:**
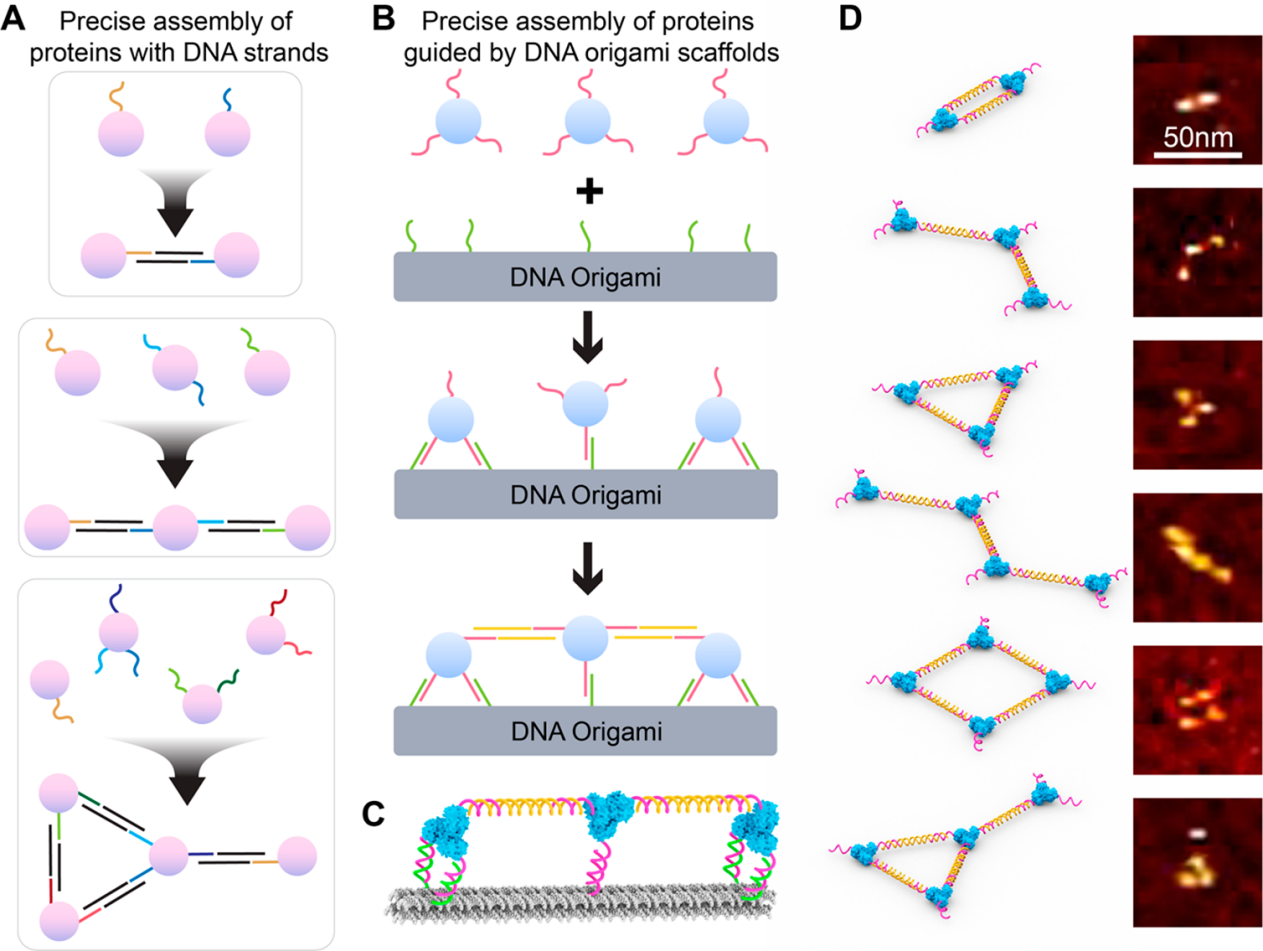
**Protein–DNA conjugate connection strategies.** (A) In the absence of a template, synthesizing a protein dimer, linear trimer, and Y-shaped tetramer requires proteins with multiple, orthogonal DNA handles, which is synthetically challenging. (B) By contrast, a DNA origami scaffold—combined with precise addition of DNA linkers and displacement strands—can generate complex shapes from a single, oligomeric protein–DNA building block. (C) A 3D model of the origami-templated trimer in (B). (D) Protein oligomers produced in this work by using DNA origami templates (top to bottom): dimer, linear trimer, triangular trimer, linear tetramer, square-shaped tetramer, and “Y-shaped” tetramer.

Here, we present a strategy that uses DNA origami as a nanoscale “assembler” to guide the linking of proteins via a series of DNA anchoring, connection, and displacement operations, forming a shape-defined protein structure (Figure 1B) that can be liberated from the origami. As a proof-of-concept, we used a homotrimeric protein–ssDNA conjugate based on a thermally stable C3 symmetric aldolase protein.^39^ By using a combination of anchor DNA strands and controlling when and how the DNA handles on the protein are connected and displaced, precise protein oligomers can be assembled on, and then released from, a DNA origami structure (Figure 1B–D). Compared to previous methods, a distinctive feature of this strategy is that it uses the same protein–DNA building blocks and DNA origami template to construct different protein oligomers. The number, arrangement pattern, and distance between proteins can all be precisely designed by the number of docking sites on the origami and the order in which the proteins are connected, which in turn makes it possible to control stepwise protein assembly into the desired shape.

The protein used in this work is 2-dehydro-3-deoxyphosphogluconate (KDPG) aldolase (ald), a 25 kDa protein that self-assembles into a C3-symmetric trimer (which we term ald_3_).^40,41^ This trimer can be approximated as a disk of 6 nm diameter and 3 nm thickness. Although ald is an enzyme, here we used it exclusively as a structural building block due to its symmetry, high thermal stability (>80 °C), and ease of expression and modification with DNA. The homotrimeric nature of the ald_3_ oligomer also allows for multiple connections and more complex assemblies (compared with a monomeric or dimeric protein). Glutamate 54, a solvent-exposed residue on the outer edge of the trimer, was mutated to cysteine (E54C) in order to perform thiol-selective chemistry with the heterobifunctional cross-linker succinimidyl 3-(2-pyridyldithio) propionate (SPDP) and a 5′-amino-modified oligonucleotide.^39^ After exposure to 6 equiv of purified SPDP modified DNA, a band with higher retention was observed by electrophoresis on a denaturing polyacrylamide gel (SDS-PAGE), corresponding to oligonucleotide-bound ald monomer.^39^ The yield is estimated to be ∼50% (Figure S1). The ald_3_-DNA trimer (Figure 2A) was purified away from trimers bearing fewer DNA strands by using anion exchange chromatography.

**Figure 2:**
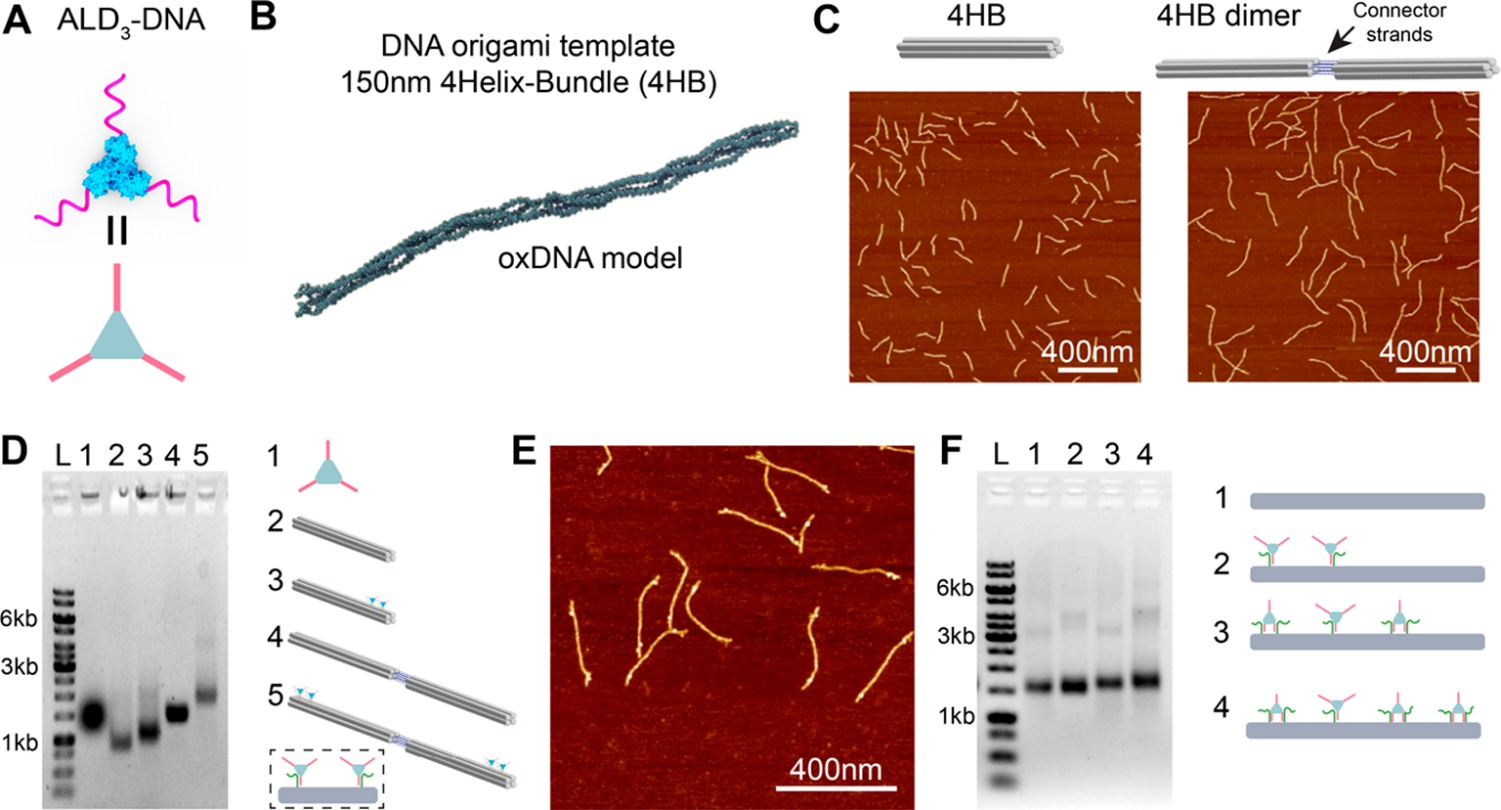
**Synthesis of ald**_**3**_**-DNA and 4HB DNA origami template.** (A) Schematic of ald_3_-DNA. The three DNA strands on the trimer are identical. (B) 4HB DNA origami. (C) AFM images of 4HB monomer and dimer. (D) Native agarose gel shows ald_3_-DNA binding to 4HB monomer and dimer. Lane L: 1kb DNA ladder. The numbered cartoons indicate the species in the corresponding well. (E) AFM image of purified 4HB dimer with two ald_3_-DNA on each origami. (F) Agarose gel of 4HB dimers with two, three, and four ald_3_-DNA. Lane L: 1kb DNA ladder. All schematic drawings show only a monomer of the DNA origami template. For simplicity, this is applied to the figures as well.

The 6 nm ald_3_ trimer is very small compared to a typical DNA origami nanostructure, which can be hundreds of nanometers in one dimension. To more readily observe the proteins tethered to the origami structure, we designed a relatively small 4-helix bundle (4HB) origami, using an 1800 nucleotide (nt) plasmid scaffold^42^ (Figure 2B, Figure S2). The 4HB is 150 nm in length and 5 nm in thickness. The small size makes it easier to see proteins arranged on the origami in the atomic force microscopy (AFM) images. The 4HB origami was assembled by mixing and annealing the scaffold (50 nM) and 5 equiv of staples in 1 × TE and 10 mM MgCl_2_ buffer. We synthesized both 4HB monomers and dimers. The latter was used in most of the experiments, for reasons discussed below. The origami was purified using 0.67% native agarose gels and imaged by AFM (Figure 2C). Then we tested the attachment of two proteins to a 4HB monomer with one attachment strand, where the distance between two proteins is 40 bp along the 4HB (Figure 2D). After purification, 10 equiv per binding site of ald_3_-DNA was added to 4HB, and the mixture was incubated at 25 °C for 48 h. Next, the sample was analyzed with a 1% agarose gel. Unfortunately, the protein–origami conjugate has a mobility very similar to that of the free ald_3_-DNA, complicating the purification process (Figure 2D). To overcome this, we engineered 4HB dimers (300 nm total length) by connecting two identical 4HBs, tail-to-tail, via four connecting strands (Figure S2). Because it has a much slower mobility than that of free ald_3_-DNA, the 4HB dimer with proteins can be successfully separated from unbound proteins (Figure 2D). Further evidence for the separation was observed in the AFM images of the purified ald_3_-4HB dimer, which showed no proteins in the background (Figure 2E). Therefore, 4HB dimers were used in all of the following experiments; however, for simplicity, all schematics depict only a single origami. Attachment of two, three, or four ald_3_-DNA onto 4HB origami dimer with either one or two attachment DNA strands was successful, showing the robustness of this approach (Figure 2F).

We next explored the optimal length of “connector” DNA: the DNA duplex for connecting ald_3_-DNA building blocks. The connector consisted of two DNA strands that form a partially complementary duplex and two identical sticky ends complementary to the ssDNA handles on the protein. We reasoned that the middle domain length should not be too long (which would increase the difficulty of protein binding) but should also be greater than 15 bp to ensure thermal stability at room temperature. We were especially concerned that—since both ends of the connectors can bind to handles on the protein—an undesired intramolecular connection might result, forming a loop on a single ald_3_-DNA conjugate and abrogating any further connections (Figure 3A). Our first test used three connectors with middle domain lengths of 15 bp, 25 bp, and 35 bp, which could bind with ald_3_-DNA modified with 21-nt handles, as reported in a previous study.^39^ We mixed ald_3_-DNA and connectors in molar ratios of 1:1.5, incubated the samples at room temperature overnight, and then analyzed them using an 8% native PAGE gel (Figure 3B). If the intermolecular connection dominates, then aggregation will occur after mixing the ald_3_-DNA and connectors. In all cases, no aggregation was observed, and the higher intensity bands indicate binding of one ald_3_-DNA with one or more connectors. The results showed intermolecular connections partially occurred, but the yield was not high enough for subsequent experiments. Shortening the connectors should increase its rigidity and therefore can potentially reduce its tendency of forming an intramolecular loop. To decrease the total length of the connector, we modified the ssDNA attached to ald_3_ from 21 to 15 nt, while keeping the middle section at either 15 or 21 bp. After incubation of ald_3_-DNA and connectors together, aggregation was observed in all ratios with the 15-bp connector (Figure 3C). The product was verified by AFM imaging (Figure S3). As expected, protein assemblies of various sizes and geometries were observed. Although we still observed some individual protein–DNA conjugates with connectors, we hypothesized that these correspond to the ald_3_-DNA binding a single connector sticky-end without forming a loop. Thus, all subsequent experiments used the 15-bp connector.

**Figure 3:**
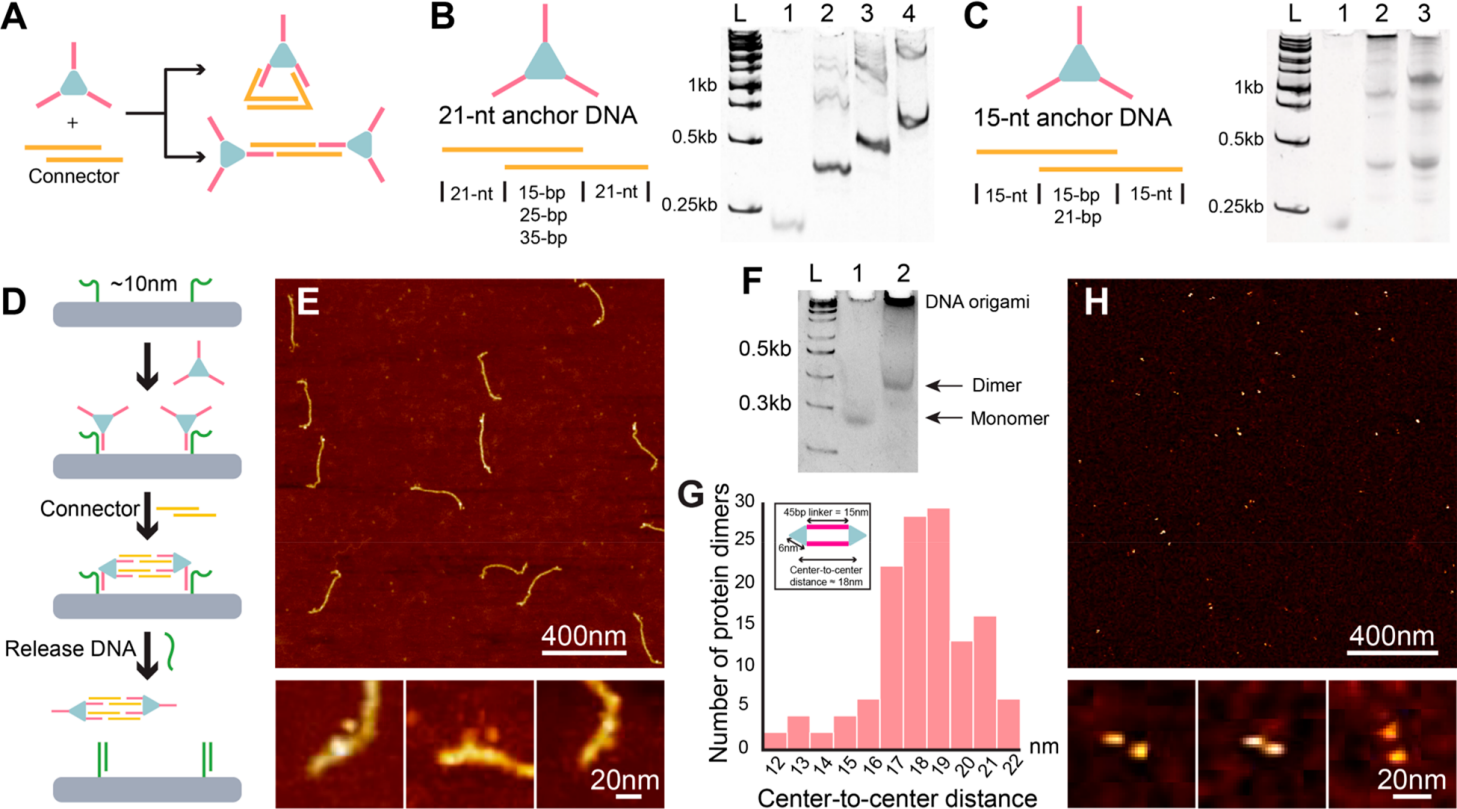
**Optimization of connector DNA and formulation of ald**_**3**_**-DNA dimer.** (A) Connector DNA can form intra- (*top*) or intermolecular (*bottom*) connections. The connector needs to be optimized to promote the desired intermolecular connection. (B) Optimization of the middle duplex region of the connector by using an ald_3_-DNA with a 21-nt ssDNA. In native PAGE: lane L, 1kb DNA ladder; lane 1, ald_3_-DNA; lane 2, ald_3_-DNA with short connector DNA (15-bp duplex in the middle); lane 3, ald_3_-DNA with medium connector DNA (25-bp duplex in the middle); lane 4, ald_3_-DNA with long connector DNA (35-bp duplex in the middle). The leading bands in lanes 2, 3, and 4 are monomers that formed an intramolecular connection. (C) Optimization of the middle duplex region of the connector by using an ald_3_-DNA with a 15-nt long ssDNA. Lane L, 1kb ladder; lane 1, ald_3_-DNA; lane 2, ald_3_-DNA with short connector DNA (15-bp duplex in the middle); lane 3, ald_3_-DNA with long connector DNA (21-bp duplex in the middle). The aggregate indicates formation of an intermolecular connection. (D) Schematic of molecular operations for dimer assembly and release on DNA template. (E) AFM images of ald_3_-DNA dimers assembled on 4HB DNA origami. (F) Native PAGE gel of ald_3_-DNA dimer after being released from 4HB. Lane L, 100-bp ladder; lane 1, ald_3_-DNA; lane 2, ald_3_-DNA dimer after release. The aggregate in the loading well in lane 2 is the DNA origami scaffold, which is too large to enter the gel. (G) Histogram of the center-to-center distance for ald_3_-DNA dimers. The inset shows the estimated distance based on the dimer structure. (G) AFM images of ald_3_-DNA dimers.

After optimization of the DNA origami template and DNA connectors, we next probed formation of a protein dimer on the 4HB template. Each attachment strand for immobilizing the ald_3_-DNA to the origami is 35 nt long—15 nt complementary to the handle on the protein and another 20 nt on the 3′ end to enable displacement of the protein (Figure 3D). Successful attachment of two proteins to the 4HB dimer was verified with AFM imaging after gel purification (Figure 3E). The ald_3_-DNA dimer can be released from the 4HB via addition of a release DNA strand, which includes a 20-nt complementary sequence to the attachment strand toeholds and a 10-nt domain hybridizing with the protein binding domain. After the final products are released from the origami, leftover connectors in solution can bind to the released protein multimer and further cause aggregation. To avoid this issue, a 15-nt “blocker” strand was added to deactivate the extra connector before the last releasing step (Figure S4). Comparing addition vs omission of the blocker strand, the products analyzed by AFM showed extensive aggregation for the ald_3_-DNA dimer without the blocker strand, confirming our hypothesis, so blocker strands were used in subsequent experiments. After being released from the 4HB template, ald_3_-DNA dimers were purified by 8% native PAGE (Figure 3F) and visualized in AFM images after purification from the gel (Figure 3G and H, Figure S5). The average distance between the two ald_3_ is 17.3 ± 0.2 nm (SD, *N* = 132), close to the calculated distance of ∼18 nm. Although the assembly of dimers on 4HB appeared to be very good, the yields of free dimers in PAGE could not be quantified, due to the trapping of DNA origami and proteins in the loading wells. As a result, we calculated the percentage of complete dimer (proteins in dimer formation/total number of proteins) after PAGE purification and obtained a yield of 84% based on AFM image analysis. This method was used for estimating the yields of other multimer formations as well.

Assembly of the ald_3_-DNA dimer was realized by anchoring each protein building block on 4HB with a single attachment DNA strand. This approach worked well for dimer formation because it allowed the maximum flexibility for connection (i.e., the protein can move more freely to accommodate the connector DNA) and because there is only one possible way to link the ald_3_-DNA building blocks. For more complex assemblies, such as the linear ald_3_-DNA trimer, the single-attachment method can lead to dimer formation and prevent subsequent linkages (Figure 4A, Figure S6). So, we used a combination of single attachment and double attachment of ald_3_-DNA to the 4HB template to eliminate unwanted side reactions (Figure 4A). After assembly and connection of the linear trimer, the product can be freed from the DNA template by adding a release DNA strand (Figure 4B). Another issue, however, arose from this new scheme: the double attachment of ald_3_-DNA is expected to limit the freedom of protein units. Therefore, it is important to optimize the interprotein distances to achieve efficient assembly. The distance between proteins along the 4HB should be large enough to prevent the “edge” proteins from binding to the handles for the “middle” ald_3_-DNA. Conversely, the distance should be small enough for the connector to link the building blocks. Two distances (40 and 32 bp) between attachment sites were tested (Figure 4C). Successful binding of three ald_3_-DNA on the 4HB for both designs was confirmed with agarose gel electrophoresis (Figure S7). Nonetheless, after adding connector DNA and release DNA, the final yields of linear trimers for the two designs are drastically different, although both the 40-bp and 32-bp interprotein distances should be shorter than the connectors. The 32-bp distance generated a clear trimer band in native PAGE, while the 40-bp sample showed a barely visible trimer band. Therefore, 32 bp was selected.

**Figure 4:**
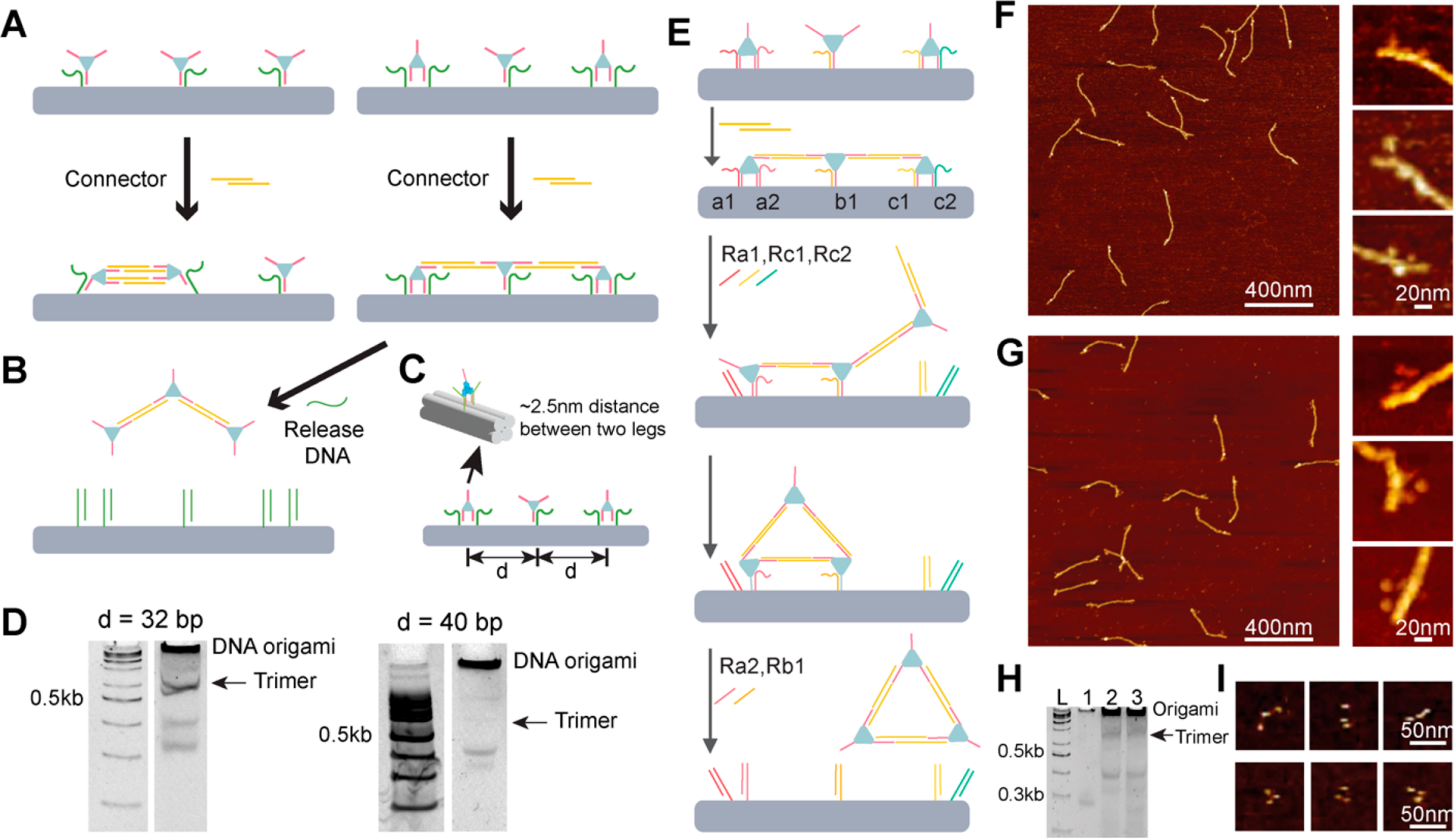
**Assembly of ald**_**3**_**-DNA trimers.** (A) Design scheme for attaching three proteins on the DNA template and connecting them to form a linear trimer. (B) Linear trimer ald_3_-DNA can be released from the DNA template. (C) Optimization of interprotein distance for more effective connection. (D) PAGE analysis of released products generated from two 4HB templates (*d* = 32 and 40 bp). Arrows in PAGE indicate the position of the ald_3_-DNA trimer. The 32-bp distance showed significantly higher yield. (E) Molecular operation scheme for forming a triangle trimer. (F and G) AFM images of linear trimers and triangle trimers on 4HB DNA origami. (H) PAGE analysis of free linear trimers and triangular timers after being released from DNA origami. Lane L, 100-bp DNA ladder; Lane 1, ald_3_-DNA; Lane 2, linear trimer; Lane 3, triangle trimer. The arrow indicates the position of ald_3_-DNA trimers. (I) Zoomed-in AFM images of linear and triangle trimers.

We next assembled a triangular ald_3_-DNA shape. Unlike the linear trimer, which was connected in a single-step reaction, constructing an ald_3_-DNA triangle requires a more complex set of molecular operations. Taking advantage of the programmability of DNA nanotechnology, we used a sequential, multistep reaction that frees up specific DNA strands at different stages to allow intended connections (Figure 4E). To realize the stepwise reactions, it is necessary to have different sequences for the attachment vs releasing strands. This orthogonality is encoded in the single-stranded toehold domains of the docking strands on the origami, allowing specific legs of the ald_3_-DNA to be released at a desired time. After forming a linear trimer on the 4HB, we released one leg of the leftmost protein (**a1**) and both legs of the rightmost protein (**c1**, **c2**). **Ald c** is thus free to swing around and bind to **ald a** by using a connector strand in solution. As a result, a triangle forms on the 4HB and can be liberated using subsequent releasing strands. AFM images confirmed both a linear trimer and a triangular trimer assembled on the 4HB template (Figure 4F and G). After release, the free trimer products were purified with PAGE (Figure 4H) and imaged with AFM (Figure 4I). After purification of free trimer products from PAGE, we estimated the percentages of complete trimers as 78% and 45% for the linear and triangular trimers, respectively (Figures S8 and S9).

Finally, we extended this method to assemble four ald_3_-DNA to three isomers: a linear ald_3_-DNA tetramer, a square, and a “Y-shaped” tetramer (Figure 1D). The percentages of complete tetramer were estimated to be 45%, 35%, and 33% for the linear tetramer, the square tetramer, and the Y-shaped tetramer, respectively. The detailed process for tetramer assembly can be found in Figures S10–S13.

To summarize, we have developed an approach that uses DNA origami as a platform, with ssDNA-modified oligomeric proteins as a common building block, and performs sequential strand displacement and reconnection operations to yield isomeric protein oligomers. Currently *de novo* protein design struggles to generate enough orthogonal interfaces to create complex protein assemblies from individual building blocks alone. Conversely, although DNA origami is a powerful technique for experimentally probing these ligand–receptor spatial interactions, a method that can directly generate these protein clusters in a DNA-minimal fashion would enable applications where origami is either not scalable or suffers from additional restrictions (e.g., degradation, stability). One application of these materials is the generation of protein ligand clusters that can best match the valence, spacing, and geometry of cell receptors to maximize bioactivity. Another application for such materials is to generate functional protein complexes *in vitro* in order to test their fundamental biology, or to generate complex biosynthetic cascades.

This work breaks from previous reports of protein–DNA conjugates, which yielded polydisperse assemblies such as nanofibers or 3D crystals, to generate unique numbers and connectivity of protein oligomers through programmatic DNA–DNA connections. More complex assemblies can be envisioned by using two populations of ald_3_-DNA, each with different DNA handles (which would allow for unique positioning on the origami). Taking the tetramer as an example, if the legs of ald_3_-DNA ***c*** have a different sequence from the ***a***, ***b***, and ***d*** building blocks, then two legs can be free for connection, and a linear tetramer can be formed in only one connection step (Figure S14). Also, in this work we demonstrated via protein complex attachment a linear, pseudo-1D origami scaffold. Much greater topological opportunities (e.g., 3D cages) are available by moving to a more complicated DNA nanostructure. Furthermore, a range of other protein oligomers with varying symmetries could be used; alternatively, developing methods for modifying a single oligomer with two or more ssDNA sequences would offer further complexity. Our approach also in principle allows for the generation of heteromultimeric protein complexes if more than one *type* of protein oligomer is used (e.g., combining ald_3_-DNA with a tetrameric aldolase^43^ modified with different DNA handles). Indeed, ultimately a series of Lego-like protein building blocks with addressable handles could be hierarchically connected through supramolecular linkages, much the way that various hybridization states of carbon (sp, sp^2^, sp^3^) are linked by organic synthesis into molecules with a complex presentation of functionality in 3D space.

Although our approach allows construction of a range of protein nanostructures from a simple set of common building blocks, additional functions can be encoded into these materials by incorporating functional proteins, either by fusing them genetically to the oligomeric building blocks or by chemical or enzymatic attachment (e.g., via SpyCatcher/SpyTag^44^) after the fact. Another benefit of this approach is that the origami mold can be reused multiple times, e.g., by attachment to a solid support, followed by centrifugation and regeneration after each round of “synthesis”. We ultimately envision applications such as multienzyme complexes performing a composite biocatalytic reaction, nanopores in biological membranes, synthetic antibodies of tunable shape and size, drug delivery vehicles, synthetic multivalent protein vaccines, and other biomolecular nanostructures.

## Supporting Information Available

Materials, design, annealing and purification protocol of DNA origami, reaction conditions and results for ald-DNA conjugation, gel electrophoresis images, and AFM images (PDF)
