# Supplementary material for: DNA-Nanostructure-Guided Assembly of Proteins into Programmable Shapes
Source: Nano Lett. 2024 Jan 26;24(5):1703–9. doi: 10.1021/acs.nanolett.3c04497 (PMC10853956; doi:10.1021/acs.nanolett.3c04497)
Supplement: Supplementary file 1 — nl3c04497_si_001.pdf [file nl3c04497_si_001.pdf]

Supplementary Information

DNA Nanostructure-Guided Assembly of Proteins into Programmable Shapes

Qinyi Lu[1], Yang Xu[2,3], Erik Poppleton[3], Kun Zhou[4], Petr Sulc[2,3], Nicholas Stephanopoulos[2,3]*, Yonggang Ke[4]*

[1]Department of Chemistry, Emory University, Atlanta, GA 30322, United States
[2]Biodesign Center for Molecular Design and Biomimetics, Arizona State University, Tempe, Arizona 85287, United States
[3]School of Molecular Sciences, Arizona State University, Tempe, Arizona 85287, United States
[4]Department of Biomedical Engineering, Georgia Institute of Technology and Emory University, Atlanta, GA 30322, United States

Email: nstepha1@asu.edu; yonggang.ke@emory.edu

Materials and methods

Materials. The single-stranded DNA scaffold (pscaf1800) was extracted from a plasmid. Chemically synthesized DNA short strands were purchased from Integrated DNA Technologies (www.idtdna.com) and were used without further purification. All other reagents were purchased from Sigma-Aldrich.

Production of custom DNA scaffold. The scaffold preparation uses a pScaf vector, which is commercially available (http://www.addgene.org/111401/). To produce the DNA scaffold, a gene with prescribed sequence should be designed first. The gene contains the desired sequence and two restriction enzyme-recognition overhangs on both ends. The pScaf vector and gene were first digested by KpnI and BamHI enzymes and purified by agarose gel electrophoresis. Then the pScaf vector and gene were mixed with 1:5 ratio and linked using T4 DNA ligase overnight. The mixtures were transformed into competent DH5α cells. The cells were grown on an LB plate containing 100 μg/mL carbenicillin, at 37˚C overnight. Three colonies were selected, and a miniprep was performed to collect the plasmids. The plasmids were then digested by KpnI and BamHI and verified by agarose gel electrophoresis. The correct insertion of the gene into pScaf was confirmed by the appearance of the corresponding bands in the gel. The recombinant pScaf plasmid and helper plasmid pSB4423 were co-transformed into competent XL1-blue cells and incubated on an LB plate containing 50 μg/mL carbenicillin and 10 μg/mL chloramphenicol, at 30˚C for two days. We selected one colony to continue the growth in 2×YT medium and collected the custom DNA scaffold following the standard protocol for extracting ssDNA.

DNA origami design and folding. The 4HB DNA origami was designed with the software caDNAno (http://cadnano.org/). For origami folding, 10 nM of the scaffold and a tenfold excess of each staple strand were mixed in 1×TE (10 mM Tris, 1 mM EDTA; pH 8.0) buffer with 10 mM $MgCl_2$. The folding mixture was annealed by heating at 80 °C and slowly cooling from 65 to 25 °C at a rate of -1 °C/5 min. Afterwards, the folded DNA origami was purified using agarose gel electrophoresis.

Agarose gel electrophoresis. DNA origami samples were subjected to agarose gel electrophoresis at 75 V for 2-3 hours in an ice water bath. Gels were prepared with 0.5× TBE buffer containing 10 mM $MgCl_2$ and 0.005% (v/v) Ethidium Bromide.

Protocol for dimer assembly. 5 equiv of connector per pair of ald_3-DNA building blocks (i.e. per dimer) was added to the sample and allowed to incubate at 25 °C overnight. Next, 4 equiv blocker strand per connector (corresponding to 20 equiv per dimer) was added, followed by incubation for 1 h. Finally, 5 equiv of releasing strands per attachment strand (10 equiv per dimer) was added for 3 h to release the dimer.

Protocol for trimer assembly. The protocol for formation of the linear trimer is the same as for the dimer. To form the triangular trimer, the linear trimer was synthesized first, then 5 equiv of releasing strands ***Ra1, Rc1,*** *and* ***Rc2*** was added, and the solution was incubated for at least 16 h for this release and re-connection process. AFM images demonstrate that the ald_3-DNA triangles formed in the expected locations (**Figure 5F**). We then added blocker strands followed by the releasing strands ***Ra2*** and ***Rb1*** to release the fully formed triangle.

Protocol for tetramer assembly. 10 equiv of connector was added to the sample and incubated at 25 °C overnight. We then added 5 equiv of releasing strands ***Rc2,*** and incubated for 16 h to enable linear tetramer formation. To release the linear tetramer, we added 40 equiv blocker and incubated the sample for 1 h, then added 5 equiv of releasing strands ***Ra1, Ra2, Rb1, Rc1, Rd1*** and ***Rd2*** for 3 h. To form the square and Y-shape, we added (to the linear tetramer still bound to the origami) ***Ra1, Ra2, Rd1, Rd2*** for the square and ***Rb1, Rd1, Rd2*** for the Y-shape, followed by another 16 h incubation. To release the square, we added strands ***Rb1*** and ***Rc1***, while the Y-shape required strands ***Ra1, Ra2*** and ***Rc1***; all strands were incubated for 3h, following an incubation for 1 h with 40 equiv of blocker strands.

Native PAGE gel electrophoresis. DNA oligonucleotides and protein samples were subjected to 8% native PAGE gel electrophoresis at 80 V for 2-3 hours at room temperature. Gels were prepared with 0.5 ×TBE buffer containing 10 mM $MgCl_2$, and stained with 1×Sybr Gold after gel running.

Extraction of protein products from PAGE. To recover a product, we cut the band and crushed it with a small pestle at room temperature. For each band, 20 µL of elution buffer (500 mM Ammonium Acetate, 10 mM Magnesium Acetate, 2 mM EDTA) was added to the crushed gel to solubilize the protein products.

AFM imaging. To visualize samples by AFM, 2 µL of the solution was deposited onto freshly cleaved mica. The sample area was then filled with 80 µL of 1×TE buffer with 10 mM $MgCl_2$. The samples were imaged on a Multimode VIII system (Bruker) in liquid using commercial tips (SNL-10, Bruker).

Protein-DNA conjugation. The ald_3 was conjugated to ssDNA handles as previously reported (Xu, Y. *et al. ACS Nano* **2019**, 13*,* 3545). All oligonucleotides were purchased with a 5' amino modifier, and functionalized with (N-succinimidyl-3-(2-pyridyldithio)propionate (SPDP), then reacted with the E54C ald mutant. The protein with three DNA handles (ald_3-DNA) was purified away from incompletely modified trimers using anion exchange chromatography, and its identity and complete modification verified by denaturing PAGE (**Figure S1**).

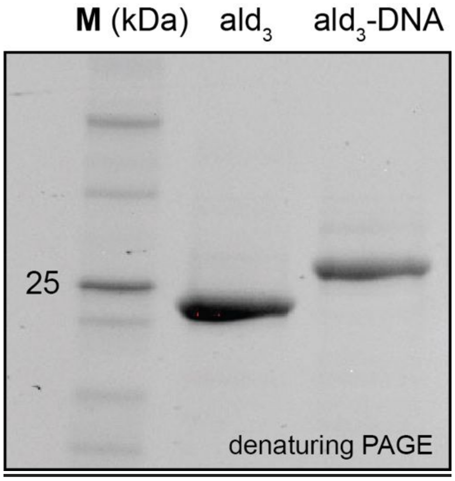

Figure S1: Denaturing PAGE analysis of the protein-DNA conjugate. The ald$_3$-DNA conjugate shows a complete band shift from the unmodified protein, confirming that all three monomers of the trimer have been successfully conjugated to DNA.

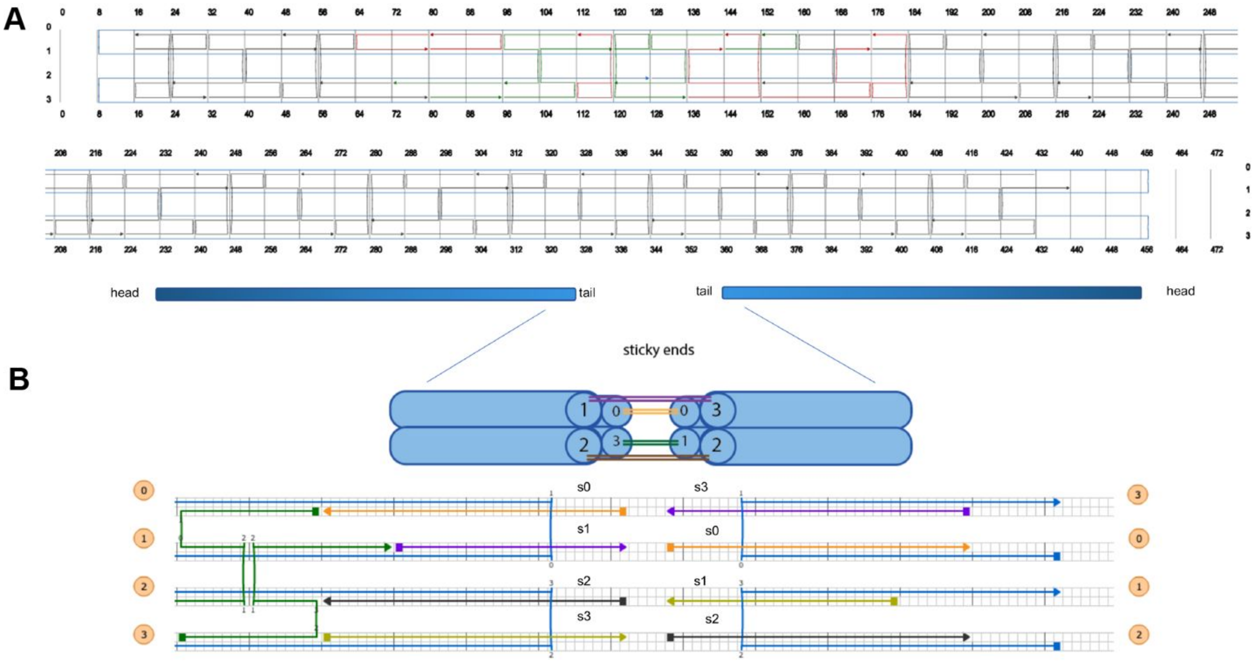

Figure S2: 4HB origami and sticky end design. (A) CaDNAno design diagram of the 4HB origami. The red strands are attachment points, which can be extended with single-stranded handles for attaching the protein. (B) Four sticky-end strands at the tail of 4HB. Sticky ends are 10 nt long. S1 is complementary to s3, s0 and s2 are palindromic (self-complementary) sequences.

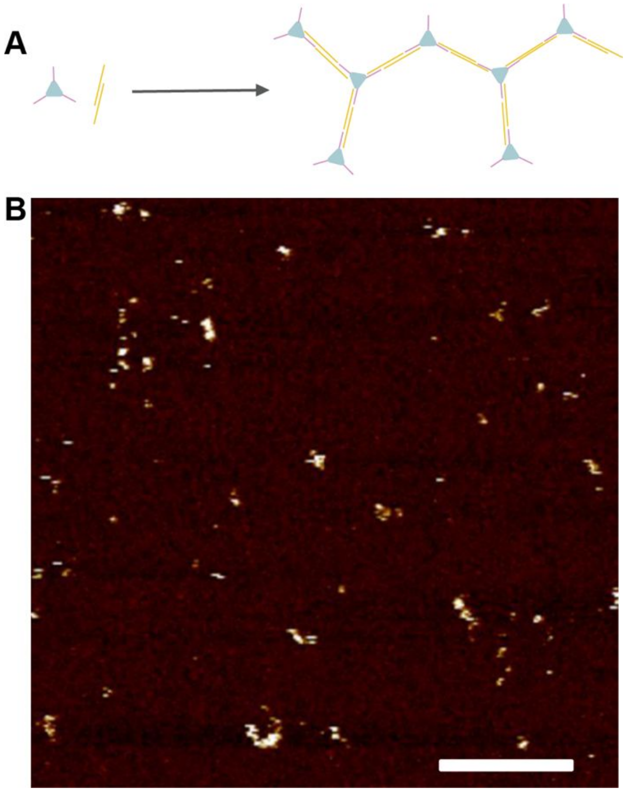

Figure S3: Connecting ald$_3$-DNA with connector DNA strands in absence of a DNA origami template. (A) Illustration of ald$_3$-DNA + connector random connection. (B) AFM image of ald$_3$-DNA + 1.5 eq connectors after 2 h. Only ill-defined, heterogeneous aggregates are observed. Scale bar: 200 nm.

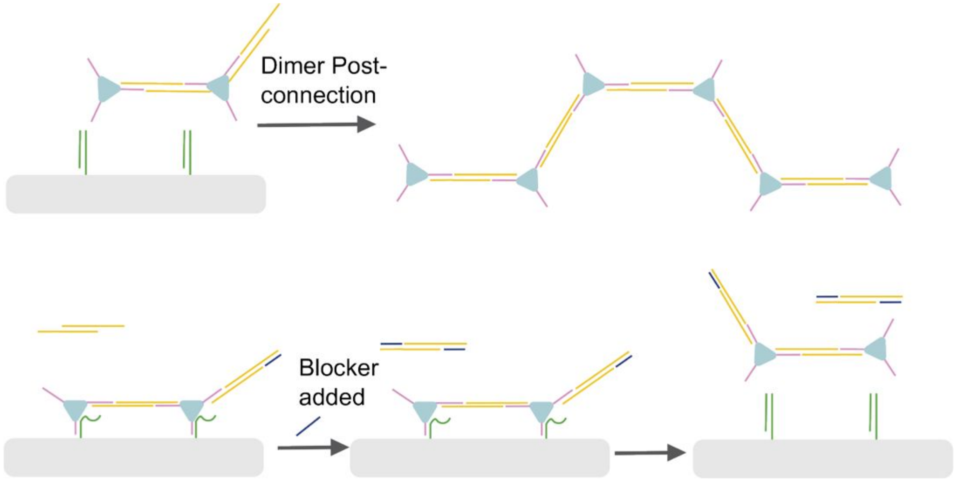

Figure S4: Deactivation of the extra connector with blocker DNA. Addition of the blocker strands prevents further spurious association of the dimer after connection (*top*), and ensures that only the desired dimer is isolated (*bottom*).

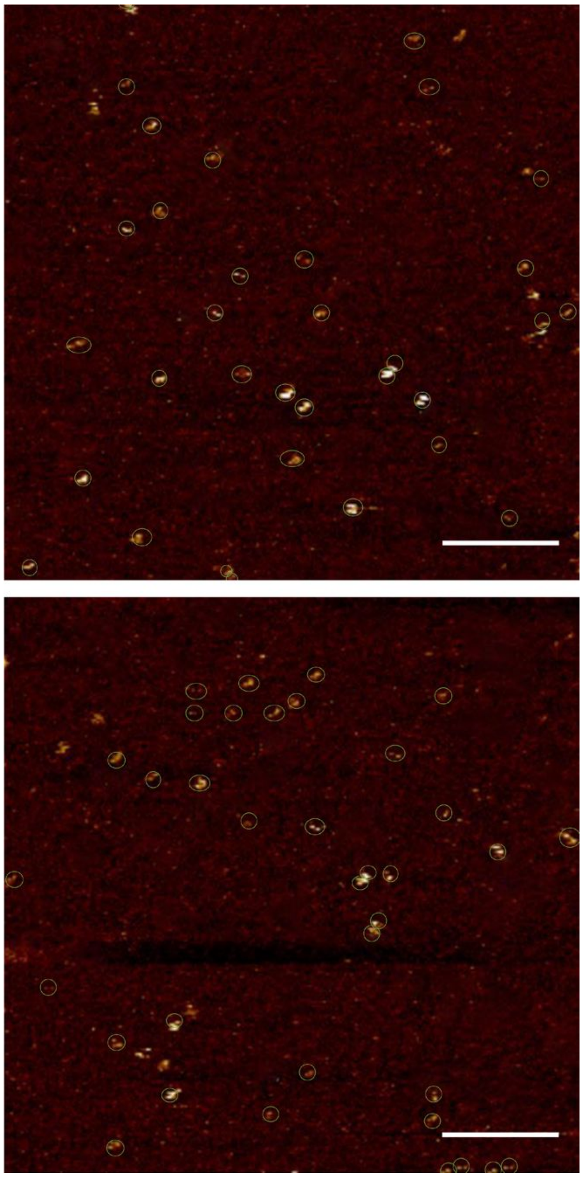

Figure S5: Wide-field AFM images of released ald$_3$-DNA dimers. Ald$_3$-DNA dimers are marked with green circles. Scale bars: 200 nm.

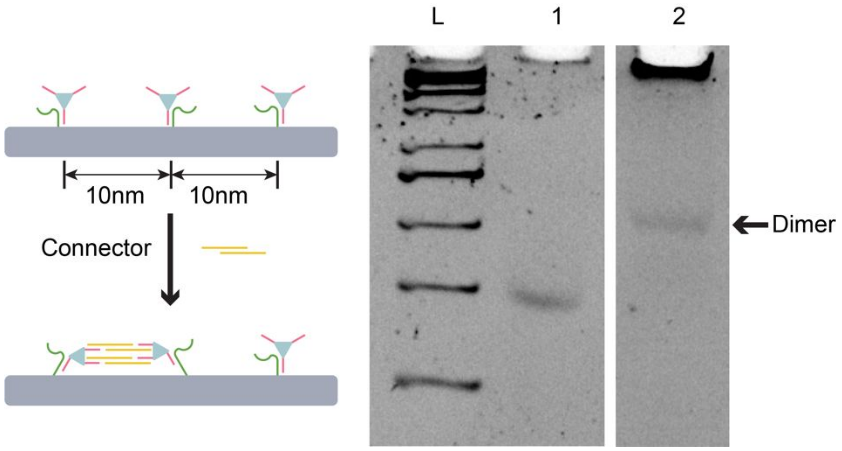

Figure S6: Connecting three ald$_3$-DNA units to 4HB with a single attachment produced only dimer products. Left: Schematic of the assembly. Right: PAGE gel of products after releasing from 4HB. Lane L: 100 bp DNA ladder. Lane 1: Ald3-DNA. Lane 2: Product. Only the dimer was observed. As a result, the leftmost and rightmost ald$_3$-DNA were attached to the origami at two points to avoid this dimer formation.

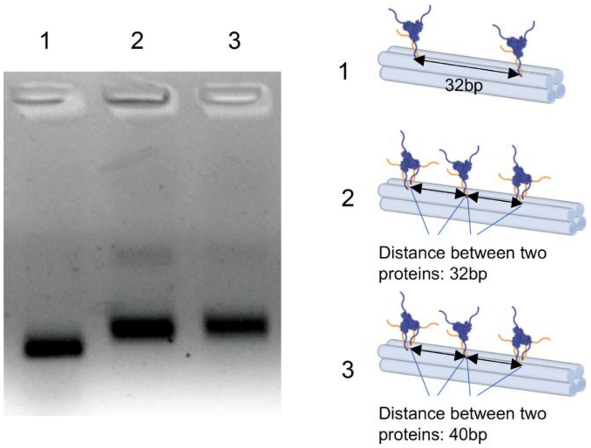

Figure S7: Agarose gel analysis of assembly of three ald$_3$-DNA on the 4HB template. Samples 2 and 3 both showed slower mobility than control Sample 1, which contains two ald$_3$-DNA, suggesting most structures of Samples 2 and 3 contain three ald$_3$-DNA.

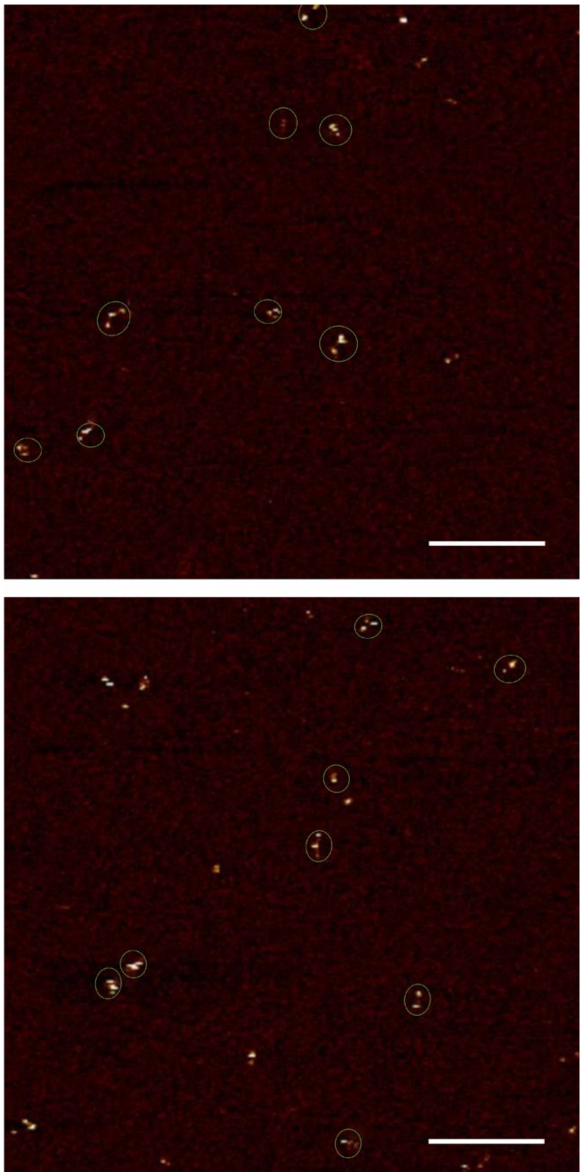

Figure S8: Wide-field AFM images of recovered linear trimer. Linear trimers are in green circles. Scale bars: 200 nm

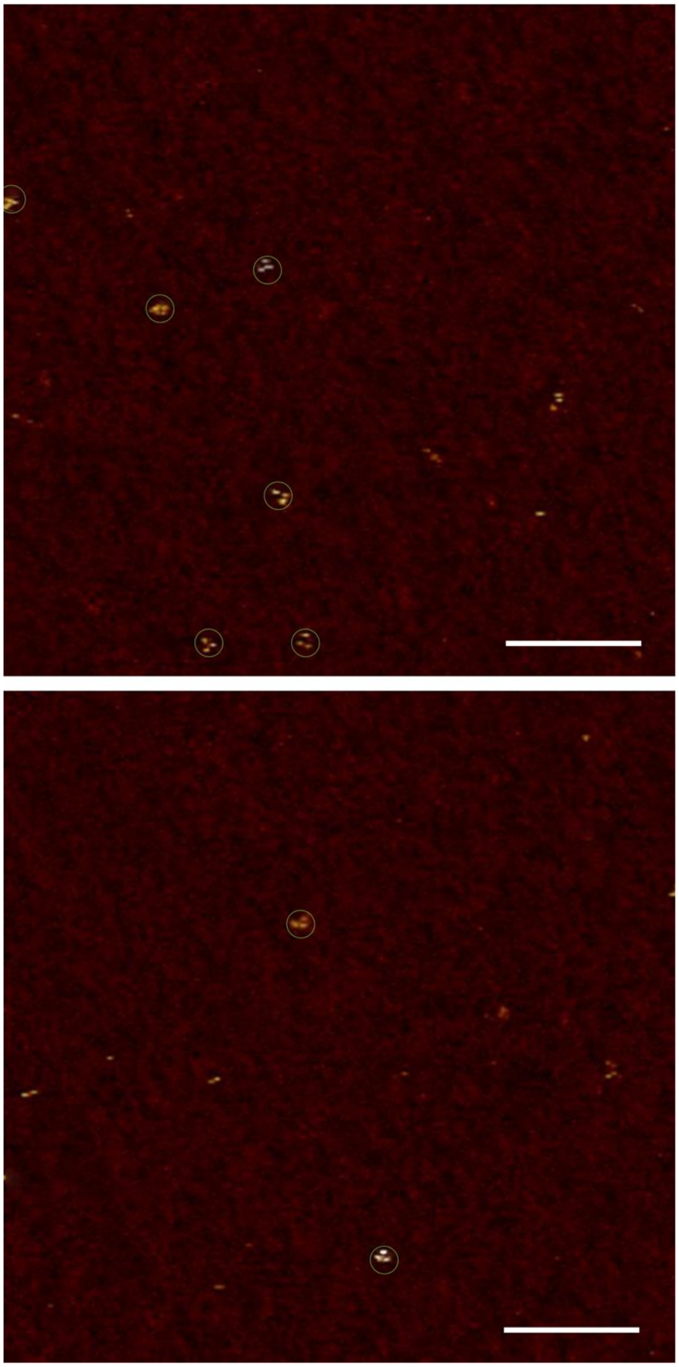

Figure S9: Wide-field AFM images of recovered triangle trimers. Triangle trimers are in green circles. Scale bars: 200 nm.

Assembly of ald$_3$-DNA tetrameric isomers:

Assemblies of four ald$_3$-DNA can produce a linear isomer and two non-linear tetrameric isomers: a square and a triangle with a single protein linked to one corner (which for simplicity we term the "Y-shape"). Both structures, however, first require assembly of a linear tetramer on the 4HB. To bind four proteins together into a linear tetramer (in the order ***abcd***) requires one free DNA leg from building blocks ***a*** and ***d*** and two legs from ***b*** and ***c***. However, ***b*** and ***c*** cannot have two free legs from the outset, or else they may form a double connection to one another, thereby preventing the formation of linear tetramers (**Figure S10A**). When arranging four ald$_3$-DNA on the 4HB, ***a, c,*** and ***d*** have two legs bound and ***b*** has only one leg bound. Two connections form after adding connectors. In one situation, ***a, b,*** and ***c*** are connected as a linear trimer, while ***d*** remains unconnected. In another case, ***a*** and ***b***, *as well as* ***c*** and ***d*** are linked as two dimers, and one leg of ***b*** is not connected to another protein. For both situations, releasing one leg of ***c*** can help it connect with the remaining legs and form a linear tetramer. At this step, adding all other six releasing strands would lead to a linear ald$_3$-DNA tetramer. However, instead of releasing the linear trimer, two assembly protocols could be implemented to make either the square or Y-shape (**Figure S10B**). With the linear tetramer on the 4HB, releasing ***a1, a2, d1, d2***, then linking ***a*** and ***d*** will result in a square. Releasing ***b1*** and ***c1*** at this point liberates the square into solution. Alternatively, releasing ***b1, d1***, and ***d2*** first, then connecting ***b*** connects with ***d***, and releasing ***a1, a2, c1*** will result in the Y-shape. It is more difficult to observe all four ald$_3$-DNA components on the origami (compared with only two or three), and the proteins are obscured and hard to distinguish given their small size and close proximity. Nonetheless, we could identify structures that clearly showed four protein building blocks on the end of 4HB (**Figure S10C**). In AFM images of the square and Y-shape, we can often directly observe the formation of the corresponding geometries formed by ald$_3$-DNA (**Figure S10C**). The native PAGE gel showed that all three tetramers have similar mobility (**Figure S10D**), and AFM imaging of the released, purified tetramers clearly showed expected formations of the ald$_3$-DNA building blocks (**Figure S10E**). The percentages of complete tetramer were estimated to be 45%, 35%, and 33% for the linear tetramer, the square tetramer, the Y-shaped tetramer, respectively (**Supplementary Figures S11** to **S13**).

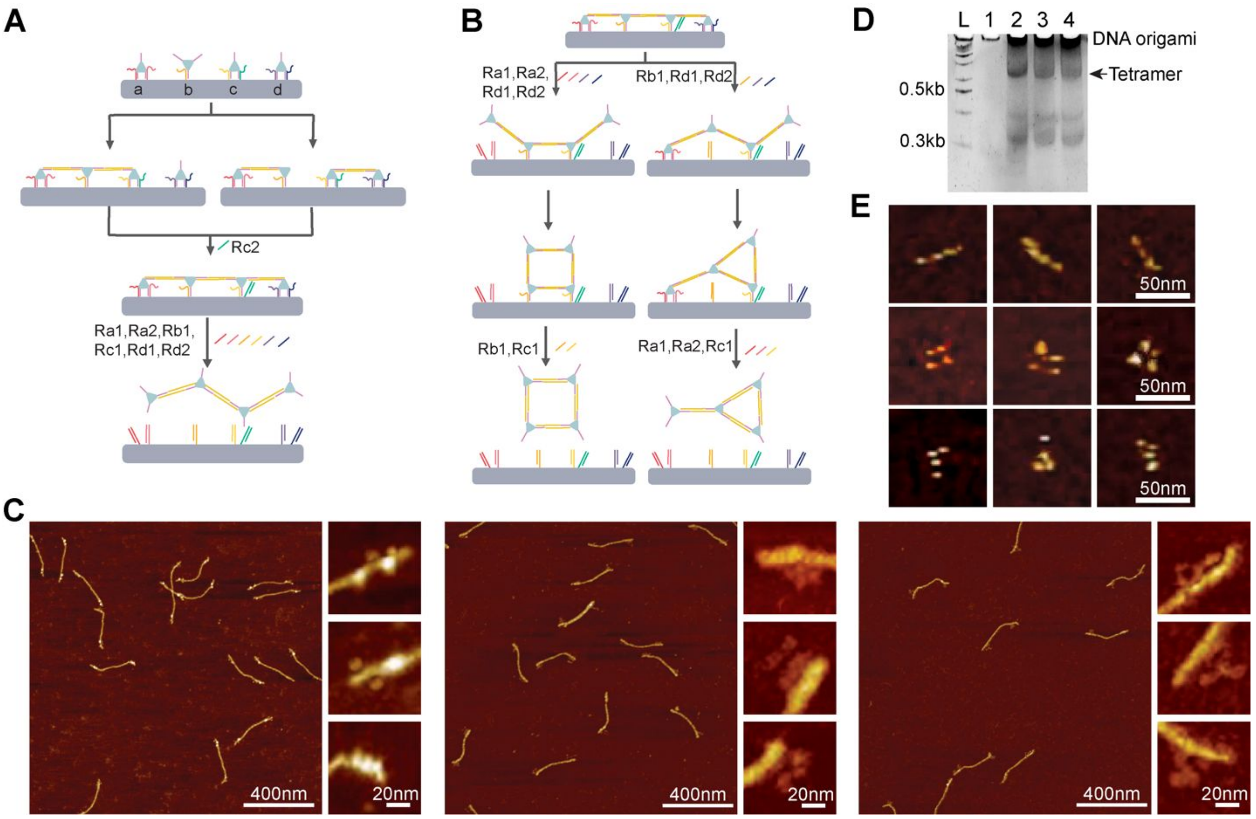

Figure S10: Assembly of ald$_3$-DNA tetramers. (A) Scheme for linear tetramer assembly. (B) Scheme for the assembly of square tetramer and Y-shaped tetramer. (C) From left to right, AFM images of linear tetramer, square tetramer, and Y-shaped tetramer on 4HB DNA origami. (D) PAGE analysis of free linear tetramer, square tetramer, and Y-shaped tetramer after being released from DNA origami. Lane L: 100 bp DNA ladder; Lane 1: ald$_3$-DNA; Lane 2: linear tetramer; Lane 3: square tetramer; Lane 4: Y-shaped tetramer. Arrows indicate the position of ald$_3$-DNA tetramers. (E) From top to bottom, zoomed-in AFM images of linear tetramer, square tetramer, and Y-shaped tetramer.

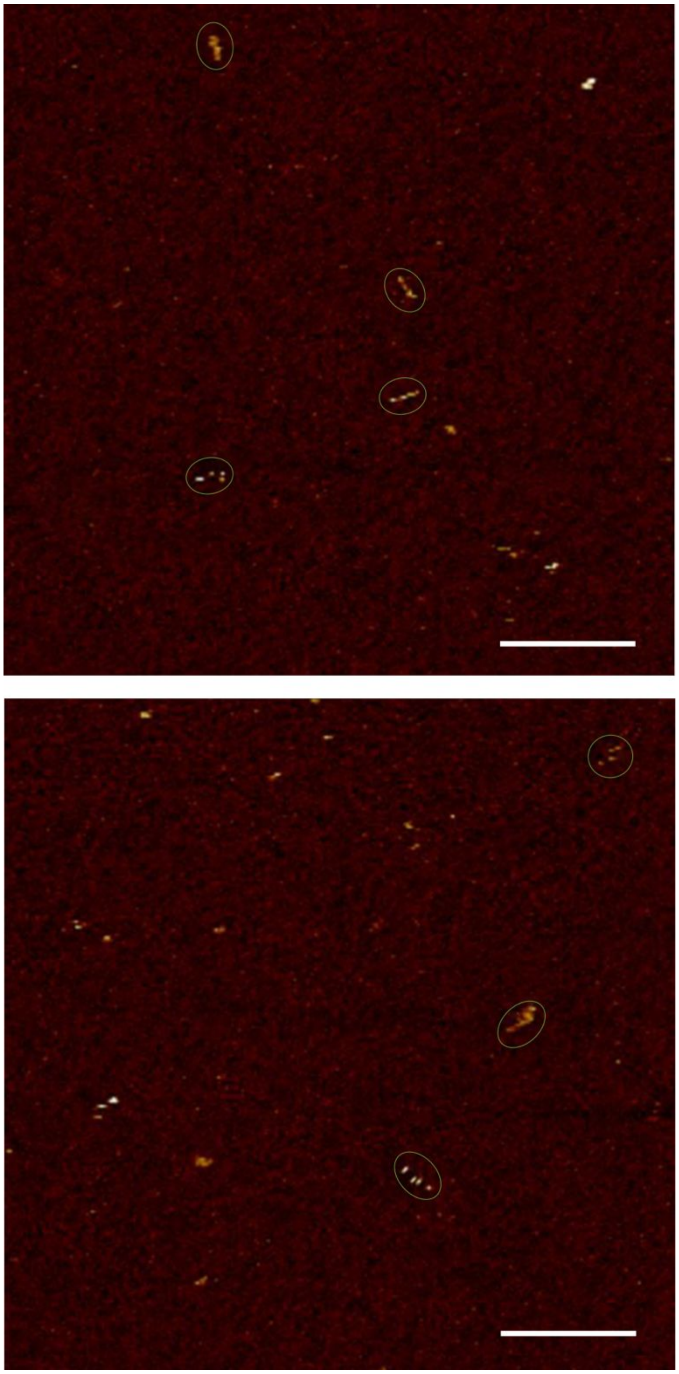

Figure S11: Wide-field AFM images of recovered linear tetramer. Linear tetramers are in green circles. Scale bars: 200 nm.

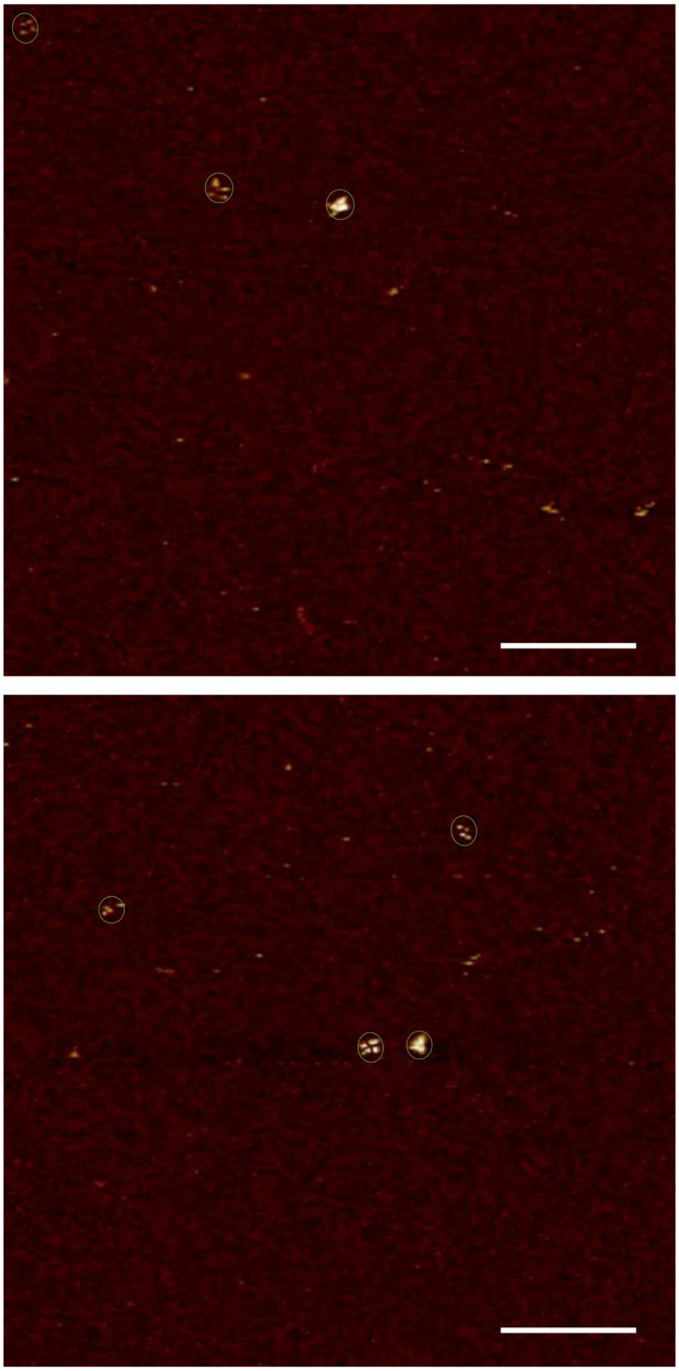

Figure S12: Wide-field AFM images of recovered square tetramers. Squares are in green circles. Scale bars: 200 nm.

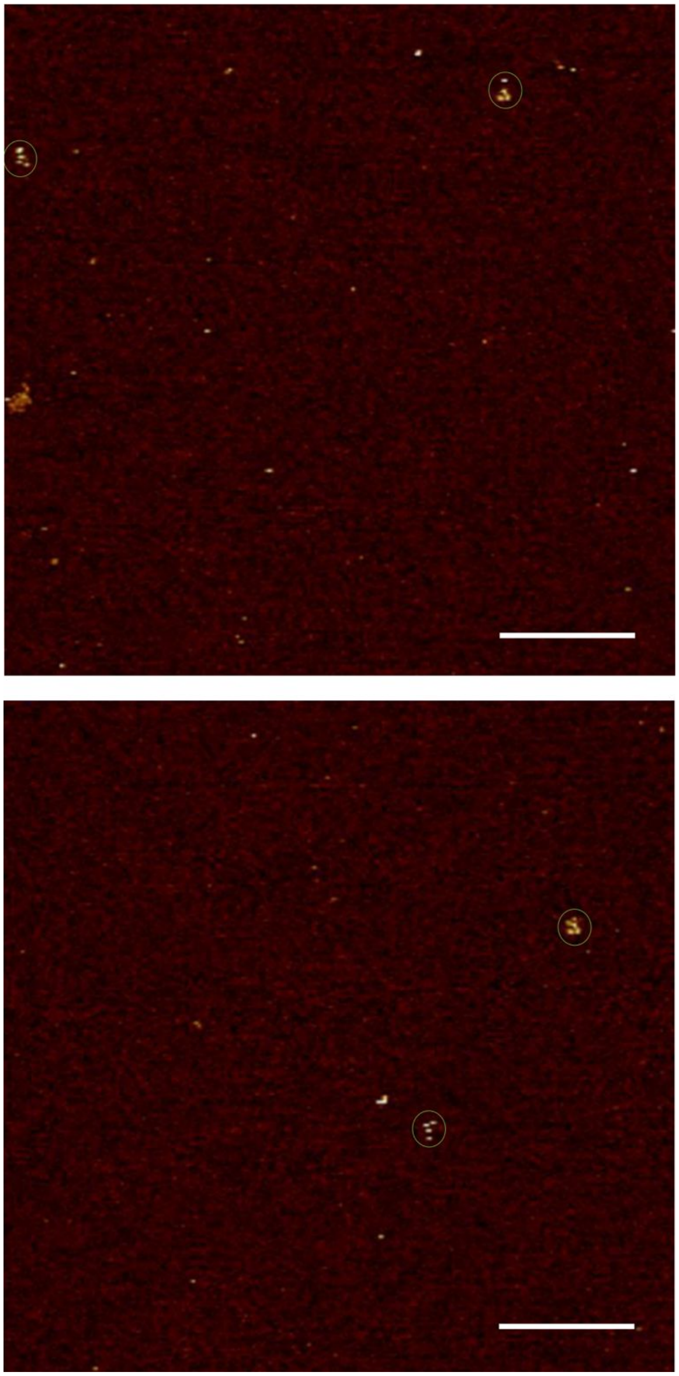

Figure S13: Wide-field AFM images of recovered Y-shape tetramers. Y-shaped tetramers are in green circles. Scale bars: 200 nm.

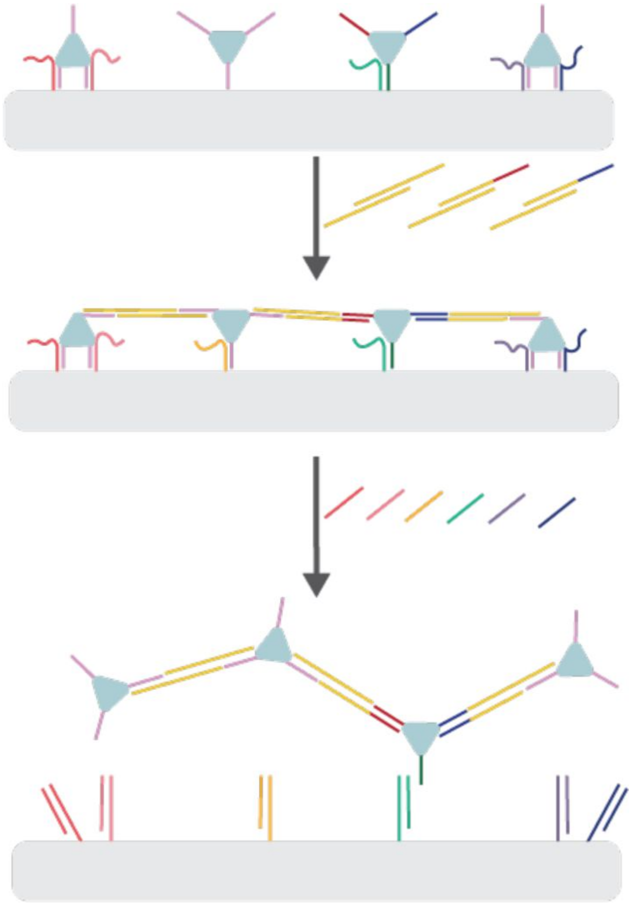

Figure S14: Assembly can be simplified by using different building blocks. For example, if an ald$_3$ c modified with three different ssDNA strands is included. Three different connectors can be added to connect four ald$_3$ building blocks in a single step to produce linear a tetramer.

Sequence of pscaf1800

AATAGTGGACTCTTGTTCCAAACTGGAACAACACTCAACCCTATCTCGGGCTATTCTTTTGATTTATAAG
GGATTTTGCCGATTTCGGGGTACCTACGAAGAGTTCCAGCAGGGATTCCAAGAAATGGCCAATGAAGATT
GGATCACCTTTCGCACTAAGACCTACTTGTTTGAGGAGTGCCTGATGAATTGGCACGACCGCCTCAGGAA
AGTGGAGGAGCATTCTGTGATGACTGTCAAGCTCCAATCTGAGGTGGGCAAATATAAGATTGTTATCCCT
ATCTAGAAGTACGTCCGCGGAGAACACCTGCCACCCGATCACTGGCTGGATCTGTTACGCTTGCTGGGTC
TGCCTCGCGGCACATCTCTGGAGAAACTGCTGTTCGGTGACCTGCTGAGAGTTGCCGATACCATCGTGGC
CAAGGCTGCTAACCTGAAAGATCTGAACTCACGCGGCCAGGGTGAAGTGACCATCCGCGAATAACTCAGG
GAACTGGATTTGTGGGGCGTGGGTGCTGTGTTCACACTGATCGGCTATGAGGACTCCCAGAGCCGCACCT
AGAAGCTGATCAAGGATTGGAAGGAGCTCGTCAACCAGGTGGGCGACAATATATGCCTCCTGCAGTCCTT
GAAGGACTCACCATACTATAAAGGCTTTGAAGACAAGGTCAGCATCTGGGCAAGGAAACTCGCCGAACTG
GACGATAATTTGCAGAACCTCAACCATATTCGCAGAAAGTGGGTTTACCTCGAACCATACTTTGGTCGCG
GAGCCCTGCCCAAAGAGCAGACCAGATTCAACAGGGTGGGCGAAGATTTCCGCAGCATCATGACATATAT
CAAGAAGGACAATCGCGTCACGCCCTTGACTACCCACGCAGGCATTCTAAACTCACTGCTGACCATCCTG
GACCAATTGCAGAGATGCCAGCGCAGCCTCAACGAGTTCCTGGAGGCGAAGCGCAGCGCCTTCCCTCGCT
TTAACTTCATCGGAGACGATGACCTGCGCGAGATCTTGGGCCAGTCAACCAATTAATCCGTGATTCAGTC
TCACCTCAAGAAGCTGTTTGCTGGTATCAACTCTGGCTGTTTCGATGAGAAGTCTAAGCACTATACTGCA
ATGAAGTCCTTGGAGGGGCAAGTTGTGCCATTCAAGAATAACGTACCCTTGTCCAATAACGTCGAAACCT
GGCTGAACGATCTGGCCCTGGAGATGAAGAAGACCCTGGAGGCGCTGCTGAAGGAGTGCGTGACAACTAG
ACGCAGCTCTCAGGGAGCTGTGGGCCCTTCTCTGTTCCCATCACAGATCTAGTGCTTGGCCGAACAGATC
AAGTTTACCGAAGATGTGGAGAACGCAATTAAAGATCACTCCCTGCACCAGATTGAGTAACAGCTGGTGA
ACAAATTGGAGCAGTATACTAACATCGACACATCTTCCGTAGACCCAGGTAACACAGAGTCCGGTATTCT
GGAGCTGAAACTGAAAGCACTGATTCTCGACGGATCCACGCGCCCTGTAGCGGCGCATTAAGCGCGGCGG
GTGTGGTGGTTACGCGCAGCGTGACCGCTACACTTGCCAGCGCCCTAGCGCCCGCTCCTTTCGCTTTCTT
CCCTTCCTTTCTCGCCACGTTCGCCGGCTTTCCCCGTCAAGCTCTAAATCGGGGGCTCCCTTTAGGGTTC
CGATTTAGTGCTTTACGGCACCTCGACCCCAAAAAACTTGATTTGGGTGATGGTTCACGTAGTGGCCAT
CGCCCTGATAGACGGTTTTTCGCCCTTTGACGTTGGAGTCCACGTTCTTT

Table S3: Staple strands for 4HB

3' End	Sequence
0[16]	GTAAAGCAAAGGGAGCGCAAGCGT
0[48]	ACCCAAATAAGCCGGCTTTCTCCA

0[80]	CACCTCAGATTGGAGCCGATGGTATCGGCAAC
0[112]	TGGACTCCCAAGTGTACTTTCAGG
0[144]	TAACCACCACACCCGCGGTCACTT
0[152]	CAATTCATCAGGCACTTCGCGGAT
0[176]	GAATAGCCCGCGTGGACACAAATC
0[200]	CCGAAATCTTTCAGCTAGCCGATCAGTGTGAA
0[240]	CCTTCCAAAGCGAGGGTTAATTGGTTGACTGGCCCAAGATCTGGGTCTCAGCTTCT
0[264]	GAGACTGATACTGCTCACCTGGTTGACGAGCT
0[304]	TAGTATGGTTGGTCCATCTCATCGAAACAGCCAGAGTTGAAATCTGGTTCAAGGAC
0[328]	GTATAGTGTCTCCACAGTCTTCAAAGCCTTTA
0[368]	CAAATTATTTGTCCTTACGTTATTCTTGAATGGCACAACTGCCAAGCATCGGCGAG
0[392]	GTTATTGGAGGGCCCATATGGTTGAGGTTCTG
1[55]	TAGAGCTTGACGGGGACAAGTTTTGGACGTACTTCTAGAT
1[79]	TCTCAGCAGGTCACCGCAATCTTATATTTGCC
1[119]	GGCGCTAGGGCGCTGGAACGTCAACATCACAGAATGCTCC
1[143]	AGTTTGGAACAAGAGTGGTCGTGC
1[175]	CGCGCTTAATGCGCCGCTACAGGGCGAGATAGAAGTAGGT
1[247]	CCGGACTCTGTGTTACCTCGCGCAGGTCATCGTCTCCGAT
1[311]	TCACCAGCTGTTACTCTACCAGCAGAGGCTGCGCTGGCAT
1[375]	AAACTTGATCTGTTCGTGCCCCTCTGGGTAGTCAAGGGCG
1[439]	TGAGAGCTGCGTCTAGCAGATCGTCGCCCACCCTGTTGAA
2[24]	GAGATGTGCCGCGAGGTTCTCCGCTTGGGGTCGAGGTGCC
2[56]	CACTACGTGAACCATC
2[72]	AAACCGTCTATCAGGGCGATGGCC
2[96]	TTAGCAGCCTTGGCCATTGACAGTAGGGCGAA
2[120]	CACCCTGGCCGCGTGACCTGAGGCCCACTATTAAAGAACG
2[152]	CAGTTCCCTGAGTTATCCTCAAACGGTTGAGTGTTGTTCC
2[184]	CACAGCACATCCAATCCCCTTATAAATCAAAA
2[216]	AGGTGCGGCTCTGGGACTGCTGGAACTCTTCGTAGGTACC
2[280]	TGCAGGAGGCATATATAACTCGTTAACAGCTTCTTGAGGT
2[344]	TTTCCTTGCCCAGATGTGCCTGCGCAAGGACTTCATTGCA
2[408]	CGAGGTAAACCCACTTGAAATCTTTCAGCCAGGTTTCGAC
3[31]	GATCGGGTGGCAGGTGCAGACCCACCCCGATT
3[79]	AGGGATAAAACAGCAGGAACGTGGCGAGAAAGGAAGGGAA
3[95]	GAAAGCGAAAGGAGCG
3[135]	TCCACTTTGTTCAGATGCGGTCACGCTGCGCG
3[207]	CTTAGTGCGAAAGGTGCCACGCCCTCCGTCGAGAATCAGTGCTTTCAG
3[223]	GGCAAAATTTCATTGGCCATTTCTTGGAATCCGTCCTCATCCAGAATA
3[271]	GAAGTTAATCCTTGATACGGAAGATGTGTCGATGTTAGTA

3[287]	ATCACGGAAAGGCGCTGCGCTTCGCCTCCAGGTGTCGCCCCAATTTGT
3[335]	CTCTGCAATGAGTCCTGCAGGGAGTGATCTTTAATTGCGT
3[351]	CTTAGACTGGATGGTCAGCAGTGAGTTTAGAACTGACCTTTCTTCGGT
3[399]	TGACGCGACGTCCAGTCTAGATCTGTGATGGGAACAGAGA
3[415]	ACAAGGGTCTTGATATATGTCATGATGCTGCGTCTGCGAACAGCTCCC

Table S4. Sequence of sticky-end strands for forming 4HB dimer

sticky 0	GACTATAGTCCAGGGCTCCGCGACCAAAGTATGG
sticky 1	TCTGGTCTGCTCTTTGAAGTCAGGCA
sticky 2	ATGGCGCCATTCCAGGGTCTTCTTCATCTCCAGG
sticky 3	TTGTCACGCACTCCTTCAGCAGCGTGCCTGACTT

Table S5. Sequences of Attachment strands and Releasing strands (Ra1-Rd2)

a1	CACCTCAGATTGGAGCCGATGGTATCGGCAACTTACCTGACGGAACTCACCGCGCCCCAGCGGGCTAGG
a2	TCTCAGCAGGTCACCGCAATCTTATATTTGCCTTACCTGACGGAACTCAGCACGGCGCCGGAGCCTGCC
b1	TGGACTCCCAAGTGTACTTTCAGGTTACCTGACGGAACTCACCCAGGCGCCCGGGGCGGCG
c1	TAACCACCACACCCGCGGTCACTTTTACCTGACGGAACTCATCCGCTACGACTTCCGGGTC
c2	AGTTTGGAACAAGAGTGGTCGTGCTTACCTGACGGAACTCACGGCGTGTCCGCGCTCGCGC
d1	GAATAGCCCGCGTGGACACAAATCTTACCTGACGGAACTCACATACGCGCGCAAGGCCGGG
d2	CGCGCTTAATGCGCCGCTACAGGGCGAGATAGAAGTAGGTTTACCTGACGGAACTCACCGGTGCGTAGCATGGTCAC
d1 fill	GAATAGCCCGCGTGGACACAAATC
d2 fill	CGCGCTTAATGCGCCGCTACAGGGCGAGATAGAAGTAGGT
Ra1	CCTAGCCCGCTGGGGCGCGGTGAGTTCCGT
Ra2	GGCAGGCTCCGGCGCCGTGCTGAGTTCCGT
Rb1	CGCCGCCCCGGGCGCCTGGGTGAGTTCCGT
Rc1	GACCCGGAAGTCGTAGCGGATGAGTTCCGT
Rc2	GCGCGAGCGCGGACACGCCGTGAGTTCCGT
Rd1	CCCGGCCTTGCGCGCGTATGTGAGTTCCGT
Rd2	GTGACCATGCTACGCACCGGTGAGTTCCGT

Table S6. Sequences of Connector strands

connector 1a	TTAAGAACCTCTCCG**GAGCAGACCTGACGGAACTCA**
connector 1b	CGGAGAGGTTCTTAA**GAGCAGACCTGACGGAACTCA**
connector 2a	TTAAGAACCTCTCCGTCGTCTGGTATAG**GAGCAGACCTGACGGAACTCA**
connector 2b	CTATACCAGACGACGGAGAGGTTCTTAA**GAGCAGACCTGACGGAACTCA**
connector 3a	AAGAACCTCTCCGTCGTCTGGTATAGATGTGAATGATG**GAGCAGACCTGACGGAACTCA**

connector 3b	CATCATTCACATCTATACCAGACGACGGAGAGGTTCTT**GAGCAGACCTGACGGAACTCA**
connector 4a	TTAAGAACCTCTCCG**ACCTGACGGAACTCA**
connector 4b	CGGAGAGGTTCTTAA**ACCTGACGGAACTCA**
connector 5a	TCGGCGCGATAGGCCGTTAGAG**ACCTGACGGAACTCA**
connector 5b	CTCTAACGGCCTATCGCGCCGA**ACCTGACGGAACTCA**

Note: The original sequence of ssDNA on the ald-ssDNA was 5' TGAGTTCCGTCAGGTCTGCTCT after decreasing the length from 21nt to 15nt, the sequence was changed to 5' TGAGTTCCGTCAGGT.
